# The Multiple Faces of C-Reactive Protein—Physiological and Pathophysiological Implications in Cardiovascular Disease

**DOI:** 10.3390/molecules24112062

**Published:** 2019-05-30

**Authors:** Magdalena Boncler, Yi Wu, Cezary Watala

**Affiliations:** 1 Department of Haemostasis and Haemostatic Disorders, Medical University of Lodz, 92-215 Lodz, Poland; cezary.watala@umed.lodz.pl; 2 MOE Key Laboratory of Environment and Genes Related to Diseases, School of Basic Medical Sciences, Xi’an Jiaotong University, West Yanta Road, Xi’an 710061, China; wuy@lzu.edu.cn

**Keywords:** C-reactive protein, inflammation, protein conformation, monomeric CRP, cardio-vascular disease

## Abstract

C-reactive protein (CRP) is an intriguing protein which plays a variety of roles in either physiological or pathophysiological states. For years it has been regarded merely as a useful biomarker of infection, tissue injury and inflammation, and it was only in the early 80s that the modified isoforms (mCRP) of native CRP (nCRP) appeared. It soon became clear that the roles of native CRP should be clearly discriminated from those of the modified form and so the impacts of both isoforms were divided to a certain degree between physiological and pathophysiological states. For decades, CRP has been regarded only as a hallmark of inflammation; however, it has since been recognised as a significant predictor of future episodes of cardiovascular disease, independent of other risk factors. The existence of modified CRP isoforms and their possible relevance to various pathophysiological conditions, suggested over thirty years ago, has prompted the search for structural and functional dissimilarities between the pentameric nCRP and monomeric mCRP isoforms. New attempts to identify the possible relevance between the diversity of structures and their opposing functions have initiated a new era of research on C-reactive protein. This review discusses the biochemical aspects of CRP physiology, emphasizing the supposed relevance between the structural biology of CRP isoforms and their differentiated physiological and pathophysiological roles.

## 1. Introduction

C-reactive protein (CRP), named for its ability to bind and precipitate the pneumococcal C-polysaccharide, is the classical acute phase protein. Although it circulates at low concentrations in healthy individuals, its levels increase dramatically in response to infections, tissue injury and inflammation [1]. The role of CRP in host defence has been thought to be largely due to its ability to bind phosphocholine (PC), activate the classical complement cascade, and enhance phagocytosis [2,3,4]. The ligand binding characteristics of CRP seem also important in understanding its role in inflammation. In addition to the recognition of microbial antigens, CRP reacts with cells at the sites of tissue injury. Similarly to serum amyloid P component (SAP), C-reactive protein binds to nuclear antigens, damaged membranes and apoptotic cells, and is involved in the clearance of injured or apoptotic cells, as well as the material released from these damaged cells [4].

In recent decades, the perception of CRP has shifted from being solely a marker of inflammation to a valuable and a very significant and independent predictor of atherothrombotic risk, including future cardiovascular events. Numerous studies have also reported that elevated CRP levels correlate significantly with the incidence of cardiovascular complications in patients without any symptoms of overt cardiovascular disease, as well as in patients with unstable angina, myocardial infarction, ischemic stroke, or peripheral artery disease. In addition, increased blood serum concentrations of CRP are regarded as a risk factor of sudden death and restenosis in patients after percutaneous coronary intervention [5].

While there is strong evidence that CRP is a predictor of arterial thrombotic events, conflicting clinical data exists on the relationship between increased plasma CRP concentration and venous thromboembolism (VTE) [6]. The great diversity of findings regarding the role of CRP in atherothrombosis has prompted the research on structures of various CRP isoforms and their possible significance in pathophysiology. The existence of modified CRP isoforms and their possible relevance to various pathophysiological conditions was suggested for the first time in the early 80s [7]. In addition, accumulating evidence indicates a need for a clear discrimination between native (larger, pentameric structure) and modified CRP isoforms (smaller, monomeric structure) and their opposing impacts under physiological and pathophysiological conditions.

As CRP has been highly conserved throughout evolution and no recognized CRP deficiencies have been discovered in humans, it is reasonable to suggest that the protein must confer a significant survival value [8], yet its precise role in human physiology and disease remains to be fully understood.

## 2. Structure of Native C-Reactive Protein

C-reactive protein (MW ~120 kDa) belongs to the family of pentraxins, proteins that have been highly-conserved over the course of phylogenesis. Pentraxins have a cyclic multimeric structure and contain ligand binding sites dependent on calcium ions. In addition, each molecule contains a flattened β-structure resembling a jellyfish, which remains distinct from other protein domains in the molecule, and which is observed in the legume lectins [9]. Structural studies of human CRP have provided a full description of the binding of CRP to phosphocholine [10,11,12,13], while structural and related studies have defined the topology and structure of the binding site for complement component C1q [14,15,16,17]. C-reactive protein consists of five identical non-covalently-bound protomers arranged in cyclic symmetry [18,19,20]. One face of each protomer (B face of CRP) presents a binding site for PC, consisting of two Ca^2+^ ions that ligate the phosphate group and a hydrophobic pocket that accommodates the PC methyl groups. On the opposite face (one of the faces of CRP), there is a deep cleft formed by parts of the N and C termini, which is bordered by an alpha helix. Mutational studies indicate that the C1q-binding site of the molecule is located at the open end of this cleft, with Asp112 and Tyr175 representing contact residues [14,21,22,23]. Several three-dimensional X-ray structures of C-reactive protein, either the free or ligand-bound form, have been deposited in the Protein Data Bank (RCSB PDB) and are freely available for research and education at PDB (http://rcsb.org) and the Molecular Modelling Database (MMDB; https://www.ncbi.nlm.nih.gov/Structure/MMDB/mmdb.shtml).

Although crystallization of CRP was originally reported in 1947 [24], forms suitable for high-resolution X-ray analysis have not been obtained yet [25]. During the years 1996-2002, at least three human CRP X-ray structures have been determined: partially calcium-loaded CRP, fully-calcified CRP (in the presence or absence of bound PC), and calcium-depleted CRP [10,11,26]. Such approaches seem to be justified by the fact that calcium ions are crucial for the binding of ligands, as well as for maintaining the integrity of the protein. In the presence of Ca^2+^, human CRP may resist denaturation induced by physical and chemical factors, such as heat, urea, and reducing agents [27,28]. The binding of calcium to CRP may also serve to protect CRP from proteolytic degradation and denaturation in circulation during the acute phase of infection [12,13,26,29]. However, physicochemical and immunological studies of the tertiary and quaternary structure of CRP have found that its structure can be modified by the microenvironment. As a result, CRP can exist in three isoforms: 1) a multimeric form composed of ten and more subunits, 2) a pentameric (native) form, and 3) a monomeric CRP form, often called “modified CRP”, which consists of a single subunit. Two of these forms, pentameric and monomeric CR, are of special interest to researchers investigating the biological function of CRP. Below, each CRP isoform will be described in detail.

## 3. Pentameric C-Reactive Protein

The most extensively studied type of C-reactive protein is its native pentameric form. Native CRP is produced by hepatocytes in response to inflammatory cytokines (IL-1, IL-6) before release into the systemic circulation. Both interleukins control expression of CRP gene through activation of the C/EBP family members C/EBPβ and C/EBPδ, which are critical transcription factors for induction of CRP. Other extrahepatic sites of CRP synthesis have been also reported (like neurons, leukocytes), although they do not substantially influence CRP plasma levels [30,31]. The native CRP has a half-life in plasma of about 19 h under physiological or pathological conditions. This interesting finding suggests that the sole determinant of circulating CRP concentration is the synthesis rate, which therefore reflects the intensity of the pathological process(es) stimulating CRP production. When the stimulus for increased production completely ceases, the circulating CRP concentration falls rapidly, to almost the same level as the CRP clearance rate [32]. It is important to note that this response by CRP to stimulus is nonspecific and is triggered by many disorders [32,33,34]. In addition, in most diseases, the circulating value of CRP reflects ongoing inflammation and/or tissue damage during the acute phase much more accurately than other laboratory parameters, such as plasma viscosity and erythrocyte sedimentation rate [32,35,36,37,38,39]. Following an acute phase stimulus, the median CRP concentration can rise by over 1000-fold from the 0.8 mg/L in healthy young adult blood donors. Subjects in the general population have stable CRP concentrations characteristic for each individual [40], without seasonal variation in baseline CRP level; however, the mean CRP value tends to increase with age [32,41].

**Figure 1:**
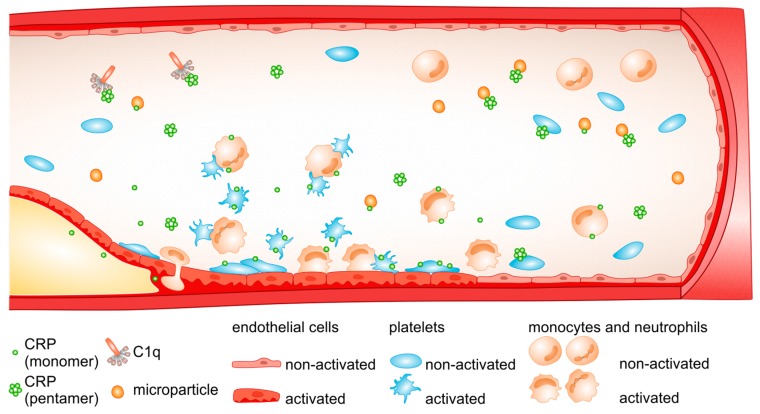
Possible interactions of CRP with blood cells and plasma components. Pentameric CRP (nCRP) bound to microbial polysaccharides or ligands exposed on damaged cells activates the classical complement pathway through the interaction with C1q. Both pro-and anti-inflammatory effects are attributed to nCRP via its interactions with blood cells (for details, please see Section 3). At the site of the injured endothelium, circulating platelets undergo activation and spread. On the surface of the activated platelets, circulating cell-derived microparticles and apoptotic cell membranes, pentameric CRP dissociates to monomeric CRP (mCRP), which exerts pro-inflammatory effects by activating blood cells and by promoting inflammatory response. mCRP can be deposited in atherosclerotic lesions.

The biological role of CRP has been clarified by studies on its structure and interactions with ligands and effector molecules. The binding sites for calcium ions and PC, the first defined ligand for CRP, are localized on the “recognition face” of CRP protomers, whereas the binding sites for the C1q complement component and Fcγ receptors are carried by the “effector face”. In addition to PC, CRP can bind to a variety of other ligands, including phosphoetanoloamine, fibronectin, laminin, chromatin, histones, ribonucleoproteins and polycations. Ligand-bound or aggregated CRP efficiently activates the classical complement pathway through direct interaction with C1q and elicits a response from phagocytic cells via interaction with the immunoglobulin receptors FcγRI and FcγRII [30,42]. In other words, by activation of classical complement pathway, ligand complexed- or aggregated CRP generates all the opsonic and pro-inflammatory effector functions of the complement system [43] (Figure 1). Ultimately, as previously found for numerous other mediators of inflammatory processes [44] CRP has pleiotropic effects on human cells, and has been found to demonstrate both pro-inflammatory and anti-inflammatory activities. The findings yielded by studies on CRP are varied, and sometimes contradictory; besides the methodological aspects, this variation has been explained by the dissociation of CRP into subunits during the experiment [45,46] and primarily by the use of low quality CRP preparations containing LPS [47], azide [48] or traces of mCRP [49].

The anti-inflammatory properties of CRP may be associated with its ability to induce the expression of interleukin-1 receptor antagonist in human peripheral blood mononuclear cells (PBMCs) [50,51,52]. Also, CRP can alter the cytokine profile by mouse macrophages by enhancing the secretion of the anti-inflammatory cytokine IL-10 and down-regulating the production of IL-12 [38,53]. When interacting with neutrophils, CRP can exert both inhibitory [38,54,55,56,57,58] or activatory effects on cell chemotaxis, degranulation or superoxide production [59,60]. Furthermore, native CRP has been shown to inhibit the respiratory burst of neutrophils as demonstrated by the extracellular release of reactive O_2_ intermediates in response to a variety of agonists such as fMLP, PAF and PMA [61]. These findings imply that CRP may play an important protective role during the early phases of the inflammatory reaction.

C-reactive protein can also have anti-atherogenic activities, as evidenced in a number of studies of platelet function which indicate that when bound to platelets, native CRP may reduce the effects of physiological platelet agonists, inhibit platelet secretion (both platelet dense body and alpha granule constituents), and decrease platelet aggregation [62,63,64,65,66,67] (Figure 1). Native CRP may also enhance the anti-platelet effect of acetylsalicylic acid [68]. It is thought that circulating CRP may play an important role in the regulation of platelet adhesion during inflammation; however, the data in this field are conflicting. For example, in patients with familiar hypercholesterolemia and coronary artery disease, native CRP increased shear-stress-dependent platelet adhesion [69], whereas in medication-free volunteers native CRP was not able to induce thrombosis by promoting platelet deposition or thrombus growth on the collagen surface [70]. In other reports, using the cone and plate system, it has been demonstrated that human, native CRP, either exogenous or endogenous, promotes platelet adhesion to endothelial cells under flow conditions (750 s-1) and this process was mediated by endothelial-derived P-selectin [71,72]. In addition, platelet adhesion to fibrinogen-coated plates has been found to be enhanced in the presence of fluid phase CRP, but only in its monomeric, recombinant form [73,74]. In similar in vitro studies on platelet adhesion to IgG- and HSA-coated cover slips, native CRP was shown to reduce platelet adhesion and moderately decrease the spread of adhering platelets as a result of CRP interactions with complement and IgG immune complexes [75]. Hence, CRP may modulate platelet response directly and indirectly via interactions with other blood constituents.

Not diminishing the importance of CRP in platelet function and thrombus formation, the concept that CRP contributes significantly to pathogenesis of atherosclerosis comes primarily from studies in which CRP preparations have been found to have pro-inflammatory and pro-thrombotic effects on vascular cells. In earlier observations, native CRP has been shown to induce pro-inflammatory cytokine release from endothelial cells (VCAM-1, ICAM-1, and E-selectin) and monocytes (MCP-1) and evoke endothelial dysfunction and monocyte adhesion to the endothelium [76,77]. However, the most compelling data implicating CRP as a determinant of endothelial dysfunction has been obtained from studies demonstrating that human recombinant CRP reduced basal and stimulated nitric oxide (NO) release from arterial and venous endothelial cells. In cultured human endothelial cells, CRP has been shown to decrease mRNA for endothelial NO synthase (eNOS), protein expression, enzyme activity (i.e., conversion of l-arginine to l-citrulline), and enzyme bioactivity (secretion of cGMP) [78,79,80,81]. These findings support previous observations of an inverse correlation between CRP concentration and endothelial vasoreactivity in patients with CAD [82]. Together with a decrease in eNOS expression and NO production, CRP has also been demonstrated to reduce angiogenesis, an important compensatory mechanism in chronic ischemia, and promote endothelial cell apoptosis in a NO-dependent fashion [79,83]. In addition, prostacyclin (PGI2), the second major vasorelaxant and anti-platelet agent present in endothelial cells, may also be downregulated by CRP, as CRP administration has been found to significantly decrease the release of PGF1α, a stable product of PGI2 from human aortic endothelial cells (HAECS) [84,85].

As a pro-coagulant, CRP is not only able to reduce PGI2 release and modulate NO metabolism in an unfavourable direction, but it may also alter the fibrinolytic system. In HAE cells incubated with CRP, time- and dose-dependent increases were observed in PAI-1 antigen concentration and activity, which was accompanied with increased concentrations of intracellular PAI-1 protein and mRNA [86]. In addition, CRP upregulated the levels of TF and PAI-1 in vein grafts, and these changes may contribute to early vein graft occlusion [87]. Similarly, it has been reported that incubation with CRP results in a reduction of the concentration and activity of endothelium-secreted t-PA, and the down-regulation of intracellular concentrations of t-PA. Moreover, it was revealed that the generation of some pro-inflammatory cytokines, like interleukin 1 (IL-1β) and tumour necrosis factor (TNFα) may mediate such t-PA inhibition [88]. Additionally, the fact that that CRP stimulates tissue factor release from mononuclear, endothelial, and smooth muscle cells [59,89,90,91] and has an inhibitory effect on the release of a natural anticoagulant—tissue factor pathway inhibitor—from human endothelial cells [92] suggests that CRP may also play a pro-thrombotic role. C-reactive protein may also increase LOX-1 expression, which plays a key role in the detrimental effects of oxidized (ox)LDL on endothelial function [93], and enhances the pro-inflammatory effects induced by angiotensin II [94]. More recently, CRP has been observed to damage the endothelial glycocalyx, further promoting the sensitivity of the endothelium towards pro-atherogenic insults [95].

It has also been shown that infusion of recombinant human CRP into healthy volunteers results in a significant increase in the serum levels of IL-6 and IL-8, serum amyloid A, serum phospholipase A2, prothrombin 1 and 2, d-dimer, and PAI-1 [96], suggesting that CRP may play a role in inflammation and the haemostatic system. Adverse effects on vascular reactivity, inflammation, and coagulation have been also reported following CRP infusion in hypercholesterolemic patients [97]. Interestingly, the CRP-mediated pro-coagulant responses observed in this study were significantly higher in hyperlipidemic patients than in normolipidemic subjects, and no change in endothelial vasoreactivity was observed in the latter group. It has been postulated that CRP infusion has a greater impact on endothelial response and coagulation in hypercholesterolemic patients than normocholesterolemic subjects [97]. A study of the triggering of coagulation cascade and inflammatory response by CRP in patients with anti-neutrophil cytoplasmic antibody (ANCA)-associated vasculitis (AAV) found that ANCA-induced netting neutrophils can prompt the formation of monomeric CRP from pentameric form, which can further accelerate thrombogenesis, enhance platelet activation and augment the inflammatory response. Therefore, an elevated concentration of CRP circulating in blood has been postulated to be not only a marker of disease activity but also a protein involved in the pathophysiology of AAV [98].

CRP and coagulation testing are commonly performed in patients presenting with secondary post-tonsillectomy haemorrhage (PTH). A study of 93 PTH patients by Biggs et al. found that these tests do not provide any additional clinically-relevant information or alter management, and as such should not be undertaken routinely in PTH patients [99].

Therefore, it is of considerable importance to clarify whether CRP plays a causative role in atherosclerosis and if so, whether it could represent an interesting novel target for intervention. Studies on drugs to specifically target and inhibit CRP effects are in progress. 1,6-bis(phosphocholine)-hexane (1,6-bis-PC) was the first CRP inhibitor found to effectively inhibit interactions between CRP and a complement by the covalent crosslinking of two CRP molecules together in the complex with a drug [100]. In rats with acute myocardial infarction (AMI), 1,6-bis(phosphocholine)-hexane reversed the effect of human CRP, resulting in the attenuation of the size of myocardium infarct and restrained cardiac dysfunction. Hence, based on this animal model, 1,6-bis-PC was proposed as a promising CRP inhibitor with cardioprotective potential in acute myocardial infarction and possible neuroprotective effects in stroke [100]. The above findings have been later supported by experimental studies indicating that 1,6-bis-PC is able to prevent the dissociation of CRP and inhibit its deposition in inflamed tissue [101]. Very recently, acetylcholine and nicotine have been found to have beneficial effects on reducing mCRP-induced inflammatory events [102]. Taken together, these findings encourage further testing: more pharmacokinetic and pharmacodynamic data acquired by local and systemic approaches are required to determine whether these substances are suitable for clinical purposes.

### 3.1. Glycosylated C-Reactive Protein

Glycosylation is a post-translational protein modification that is involved in most physiological and disease processes. The oligosaccharides are covalently bound to the proteins through the nitrogen or oxygen atoms of amino acids, forming N- and O-linked glycoproteins. Both N- and O-linked protein glycosylation have implications on protein stability, structure, molecular recognition and intracellular trafficking [103,104,105,106,107]. In inflammatory diseases (mainly in chronic inflammatory conditions), numerous changes in the glycosylation of serum proteins, such as α1-acid glycoprotein, immunoglobulins, transferrin, haptoglobin and α2-macroglobulin have been reported, as well as their effects on protein function [108]. However, with regard to C-reactive protein, only isolated reports have examined protein glycosylation [109]. This may be due to the fact that human CRP is normally a non-glycosylated protein. In addition, its half-life is relatively short (19 h) compared to the most human plasma proteins [110], which may raise a question about the likelihood of CRP glycosylation. Nevertheless, Das et al. [109] report that under some pathological conditions, CRP can undergo this phenomenon. Using CRP purified from the samples taken from patients with systemic lupus erythematosus (CRPSLE), acute lymphoblastic leukaemia (CRPALL), tuberculosis (CRPTB), visceral leishmaniasis (CRPVL), osteogenic sarcoma (CRPOS) and Cushing’s syndrome, the authors demonstrated the presence of sugars such as sialic acid, glucose, galactose, mannose in CRPs, and revealed differences in CRP carbohydrate and amino acid composition between different samples. In addition, molecular modelling suggests that there are two potential glycosylation sites on the cleft floor of CRP molecule, which can be opened up after slight changes in the protein sequence, without affecting other functional areas of the petraxin structures [109].

Some of those examined molecular variants have been further characterized based on the binding of antibodies to CRP and the interactions with some plasma glycoproteins (IgG, fibronectin, fetuin and asialofetuin) and cells [111,112]. Five CRP variants have typically been used for the antibody-binding experiments: CRPSLE, CRPALL, CRPTB, CRPVL, and CRPOS. All of these variants displayed differential levels of binding to antibodies, fibronectin and human IgG. For example, at the highest CRP concentration (1 µg), CRPVL displayed the highest level of binding to fibronectin, which was more than twice that observed with CRPALL. The degree of anti-CRP binding differed in the following order: CRPALL > CRPVL > CRPOS > CRPTB/CRPSLE [111].

As anaemia is a common manifestation in tuberculosis and visceral leishmaniasis, Ansar et al. [112] assessed the contribution of glycosylated CRPTB and CRPVL in haemolysis of erythrocytes in patients with TB and VL. Two glycosylated CRP variants have been shown to bind several times more readily to diseased erythrocytes than to the cells obtained from healthy subjects. Alterations in cell membrane fragility, fluidity, and hydrophobicity, eventually leading to haemolysis via complement activation were observed in erythrocytes from patients following binding of CRPTB and CRPVL. Thus, these observations indicate that disease-associated CRP is able to efficiently trigger a complement pathway and provide a possible mechanism for hemolysis causing anaemia in patients with TB and VL [112].

### 3.2. High-Order Assembly of Pentameric C-Reactive Protein

To date, several reports have suggested the existence of human C-reactive protein in a multimeric form. The fibril-like structures of CRP were first reported by Wang et al. [113] by the combination of size-exclusion chromatography and electron microscopy. These fibrils were composed of face-to-face stacking pentameric CRP molecules, which were formed rather slowly over the course of days. The amount and length of the fibrils observed on membranes was dependent on the ionic strength of the solution. When the ionic strength was low, more fibrils composed of larger numbers of repeating units could be observed on charged lipid membranes, implying that the fibrils occur in solution due to electrostatic forces between pentameric rings which adsorb onto membranes by non-specific interaction [113].

A study of the structure of CRP multimers based on analytical ultracentrifugation (AUC) has revealed the presence of larger CRP structures in solution [114]. A buffered solution of highly purified CRP was found to contain primarily pentameric native CRP (MW 120 kDa), but also included the 241 kDa CRP decamers, which most likely were formed through intermolecular associations of CRP pentamers. Moreover, it has been established that the share of CRP multimers in solution reaches about 10% in the presence of 2 mM CaCl2 and this may further increase after the removal of calcium ions [114]. The presence of decameric CRP in solution and on surfaces has also been confirmed using a combination of different analytical instrumentation methods [115]. It has been suggested that in a physiologically-relevant buffer, native CRP exists in a rapid pentamer-decamer equilibrium. However, in contrast to the earlier observations, it has been shown that the non-physiological removal of calcium ions can reduce the proportion of decamers, which is accompanied by a slow dissociation of CRP into protomers. At present, the decamer form of CRP is not known to have any direct function, although a role in host defence or apoptotic cell clearance cannot be ruled out: the rapid equilibrium between the CRP pentamer and its decamers provides a way to reduce non-specific protein binding to CRP, thus maintaining the integrity of CRP when it is abundant in plasma [115]. This mechanism may be functionally important, because plasma is a highly complex mixture of the order of thousands of different proteins. Further studies are needed to establish whether pentamer-decamer exchanges occur in blood. Crystallographic studies of calcium-depleted human CRP have attributed the decameric structure of CRP to interactions between two CRP A faces of two independent pentamers [26]. If so, it can be assumed that the presence of decamers may have an effect on the interactions between CRP and ligands, which are presumed to bind to the A face of CRP; conversely, the interactions of native CRP with ligands, for example factor H, may also limit formation of CRP decamers [115].

Recently, in addition to the pentameric isoform, CRP has been detected as a trimer and a tetramer in blood of patients with cardiovascular disease, as well as in the blood and other tissues of human CRP transgenic rats [116]. However, the existence and physiological role of these CRP isoforms is unclear and requires further investigation.

## 4. Monomeric C-Reactive Protein

It has long been known that pentameric CRP can spontaneously, yet slowly dissociate into subunits in vitro, forming monomeric CRP (mCRP) in calcium-free buffers at mild alkaline [20] or physiological pH [117]. Neutral denaturant (i.e., urea) or heating greatly accelerates the dissociation process, but their effects again require the absence of calcium [27,118]. So far, only highly-charged denaturant (i.e., guanidine hydrochloride) or strong acidic pH appears to be able to dissociate pentameric CRP, irrespective of the presence of calcium [118]; this is most likely due to their ability to disrupt the electrostatic interactions that mediate calcium binding to CRP. As body fluids usually contain high levels of calcium, it has been suggested that the dissociation of pentameric CRP is unlikely in vivo [32]. However, early clues suggesting that pentameric CRP may dissociate in vivo were obtained from observations on lipid monolayer-bound CRP by negative-stained electron microscopy (EM) [119,120]. Dissociated subunits were observed following the incubation of lipid monolayers with CRP in the presence of calcium. The authors suggest that calcium-dependent binding of pentameric CRP to damaged cell membranes could induce this dissociation, probably leading to the formation of mCRP [120]; however, the resolution of negative-stained EM (~20 nm) used in these studies was limited, and calcium-free purification and storage buffers were used, which may have increased the chance of spontaneous dissociation for the pentameric CRP [119,120]. Indeed, identical patterns of subunit arrangement on lipid monolayers have been observed using purified, spontaneously-dissociated CRP subunits [121]. Finally, the question of whether mCRP was ultimately formed following calcium-dependent binding of pentameric CRP to damaged membranes remains to be formally tested.

Biophysical, antigenic and functional assays on highly-purified proteins indicated that calcium-dependent binding of pentameric CRP to lipid monolayers mimicking damaged cell membranes results in the formation of dissociated subunits with a partially retained native conformation; this process eventually results in the formation of mCRP [122]. Although the pentameric CRP was also found to dissociate on apoptotic cell membranes, this required the generation of lysophosphatidylcholine [122]. Similar in vivo dissociation conditions have been observed for activated platelets [48,123,124], necrotic cell membranes [15,101,125,126,127], acidic pH [128], oxidative stress [129,130], microparticles [131], amyloid plaques [132] and neutrophil extracellular traps [98] (Figure 1). All the above data underline the important role played by posttranslational modifications of the CRP molecule in the modulation of its pro-inflammatory activity.

Interestingly, most of the aforementioned dissociation scenarios that generate mCRP are specifically associated with inflammation. It is thus plausible that mCRP should be predominately generated locally within lesions. A recently-proposed model suggests that the generation of mCRP in human inflamed tissue begins with the binding of native CRP to activated monocytes, which is subsequently released on microvesicles. On binding to these microvesicles, CRP may dissociate into subunits giving mCRP or undergo structural alterations without disrupting the pentameric symmetry; these enable the activation of a complement system and the promotion of inflammatory response by the activation of endothelial cells and leukocyte recruitment to the injured tissue [15]. Given the enhanced pro-inflammatory activities of mCRP, it is possible that besides being an activating mechanism, the conversion of pentameric to monomeric CRP also serves as a buffering mechanism that localizes the pro-inflammatory actions at inflammatory loci [122]. This mechanism could protect the body from systemic challenge in response to large rises in the circulating levels of pentameric CRP. Of note, mCRP have been shown to be demonstrate opposing or overlapping bioactivities with pentameric CRP, e.g., activation of complement [126,127,133,134], stimulation of endothelial cells [28,46,135], neutrophils [136,137,138,139] and platelets [48,70,129], and binding to ligands including low-density lipoprotein [45], C1q and CFH [133,140] (Figure 1). This suggests that at least some of the reported actions of pentameric CRP may actually derive from the mCRP formed during purification or storage.

Although these dissociation scenarios suggest that mCRP can be generated in vivo, it is difficult to obtain definite proof. Most previous efforts rely on the distinct antigenicity expressed by mCRP to detect its in vivo generation by immunohistochemical staining [28,124,141], flow cytometry [131,142], or intravital imaging [15,101]. However, the antibodies used in these studies either cross-react with both pentameric and monomeric CRP (clone 8) [143], or recognize an epitope also exposed by pentameric CRP with reversible or moderate conformation changes (i.e., 3H12/9C9 against amino acids 199-206) [10,15,122,125,128,144,145]. Sample preparation procedures, e.g., fixation/antigen retrieval in immunohistochemical staining, may also artificially change the conformation of CRP and hence complicate the interpretation of the result. Definite evidence for the in vivo generation of mCRP could be obtained by the identification of autoantibodies that specifically react with epitopes unique to mCRP [146,147,148]. Fortunately, this goal has been achieved by identifying an autoimmune epitope in lupus nephritis (LN) that is exposed only in mCRP, i.e., amino acids 35-47 [28,149]. The strong association of this autoimmune epitope with the prognosis of LN patients further suggests that an etiological role is played by mCRP.

The context- and conformation-dependent actions of CRP may address the enduring question of how CRP, a major acute protein, can act as a fine regulator of inflammation [32]. Moreover, the different localization and activities of distinct CRP conformations may also account for its varied phenotypes in animal models [150,151,152,153,154,155,156] and elusive causal relationship with diseases [157,158,159]. Given that mCRP might be the major conformation acting in lesions, treatment may be achieved by targeting it both therapeutically and diagnostically. In this regard, inhibitors either preventing the dissociation of pentameric CRP [15] (described in Section 3) or blocking the pro-inflammatory actions of mCRP [140,149,160] have been developed. With regard to the latter, an amino acid sequence called CBS (cholesterol binding sequence) has recently been identified in the mCRP molecule; CBS is believed to be responsible for the binding of ligands by the protein. It has been found to have the potential to inhibit the binding of mCRP to various ligands (apolipoprotein B, cholesterol, C1q, fibronectin, collagen and fibrinogen) and reduce the pro-inflammatory effects of mCRP on leukocytes and endothelial cells [140]. In addition, CBS has been shown to potentiate factor-H cofactor (CFH) activity [149], as well as regulate mCRP-dependent osteoclast differentiation [160]. Overall, these results strongly suggest that CBS is an effective inhibitor of in vitro and in vivo effects of mCRP, and may be a promising new tool for the development of anti-CRP therapeutic strategies. Moreover, circulating or microparticle-bound mCRP has been found to be a better diagnostic index than pentameric CRP in myocardial infarction [131,161] and peripheral artery disease [142] using homemade assays; for example, an ELISA assay designed for quantifying plasma mCRP based on commercially-available reagents [162] may be a promising tool for evaluation of mCRP in plasma. This sort of assay is a “gold standard” by which protein biomarkers are validated and monitored in diagnostic laboratories. However, each diagnostic evaluation of a ligand binding assay requires antibodies specific to a candidate biomarker, and no antibodies specific for mCRP are currently commercially available; in addition, the accuracy, reproducibility, sensitivity, and specificity of the test require analytical validation according to diagnostic guidelines [163].

## 5. Conclusions

The reliable and successful study of the role of CRP in vivo is a challenging area for researchers. It requires a good knowledge of the chemical structure and functionality of the protein, as well as its physical properties, a comparison of the effects of existing CRP forms and a good familiarity with the field of research. Although it is essential to use only highly-purified and properly characterized CRP preparations, a number of commercial CRP preparations are generated in E. coli and are hence contaminated with bacterial products; they also contain high levels of azide, which are known to produce pro-inflammatory effects in the biological system when they are not removed from CRP solution. This variation in CRP reagent quality could be one of the main reasons for the presence of such contradictory and confusing empirical findings associated with its use [47,78,96,164,165,166].

Furthermore, attempts to define the role of C-reactive protein face a number of methodological obstacles resulting from the lack of commercially-available antibodies recognizing CRP monomers (see above) or problems presented by the biological features of CRP. Among the latter is a tendency of CRP molecules to change their structure and function upon immobilization on polystyrene plates [118] or the surface of sensors [17]. In addition, CRP monomers display reduced solubility compared to native CRP [165,167].

Another important issue is associated with finding reliable and effective ways to examine mechanisms of mCRP interactions with cells. One typical approach based on blood platelets is beset by three potential interactions: (i) CRP may affect platelet response via receptor and non-receptor mechanisms, (ii) at least a few receptors on platelets, such as CD36, GPIIbIIIa and GPIb-IX-V may be involved in the regulation of platelet function by CRP and (iii) monomeric CRP has a propensity to bind to immunoglobulins [168,169].

Studies on monomeric CRP typically address the effects of Cys-mutated recombinant mCRP, which lacks the capacity to form disulphide linkages (reduced mCRP) [165]. Although such observations are justified in the light of the role played by intra-subunit disulphide bridges in the regulation of CRP function in vivo, these studies should be balanced with those of monomeric CRP with the intact primary structure (non-reduced CRP). The vital question about the catabolism of CRP monomers, raised many years ago, still remains open.

## References

[1] Wu Y., Potempa L.A., El Kebir D., Filep J.G. (2015). C-reactive protein and inflammation: Conformational changes affect function. Biol. Chem..

[2] Pepys M.B. (1981). C-reactive protein fifty years on. Lancet.

[3] Gewurtz H., Mold C., Siegel J., Fiedel B. (1982). C-reactive protein and the acute phase response. Adv. Intern. Med..

[4] Du Clos T.W. (2003). C-Reactive Protein as a Regulator of Autoimmunity and Inflammation. Arthritis Rheum.

[5] Ridker P.M. (2003). Clinical application of C-reactive protein for cardiovascular disease detection and prevention. Circulation.

[6] Lippi G., Favaloro E.J., Montagnana M., Franchini M. (2010). C-reactive protein and venous thromboembolism: Causal or casual association?. Clin. Chem. Lab. Med..

[7] Simpson R.M., Prancan A., Izzi J.M., Fiedel B.A. (1982). Generation of thromboxane A2 and aorta-contracting activity from platelets stimulated with modified C-reactive protein. Immunology.

[8] Nillson J. (2005). CRP-marker or maker of cardiovascular disease?. Arterioscler Thromb. Vasc. Biol..

[9] Emsley J., White H.E., O’Hara B.P., Oliva G., Srinivasan N., Tickle I.J., Blundell T.L., Pepys M.B., Wood S.P. (1994). Structure of pentameric human serum amyloid P component. Nature.

[10] Thompson D., Pepys M.B., Wood S.P. (1999). The physiological structure of human C-reactive protein and its complex with phosphocholine. Structure.

[11] Shrive A.K., Cheetham G.M., Holden D., Myles D.A., Turnell W.G., Volanakis J.E., Pepys M.B., Bloomer A.C., Greenhough T.J. (1996). Three dimensional structure of human C-reactive protein. Nat. Struct. Biol..

[12] Goda T., Miyahara Y. (2018). Specific binding of human C-reactive protein towards supported monolayers of binary and engineered phospholipids. Colloids Surf. B Biointerfaces.

[13] Goda T., Miyahara Y. (2016). Engineered zwitterionic phosphorylcholine monolayers for elucidating multivalent binding kinetics of C-reactive protein. Acta Biomater..

[14] Agrawal A., Shrive A.K., Greenhough T.J., Volanakis J.E. (2001). Topology and structure of the C1q-binding site on C-reactive protein. J. Immunol..

[15] Braig D., Nero T.L., Koch H.G., Kaiser B., Wang X., Thiele J.R., Morton C.J., Zeller J., Kiefer J., Potempa L.A. (2017). Transitional changes in the CRP structure lead to the exposure of proinflammatory binding sites. Nat. Commun..

[16] McGrath F.D.G., Brouwer M.C., Arlaud G.J., Daha M.R., Hack C.E., Roos A. (2006). Evidence That Complement Protein C1q Interacts with C-Reactive Protein through Its Globular Head Region. J. Immunol..

[17] Biro A., Rovo Z., Papp D., Cervenak L., Varga L., Füst G., Thielens N.M., Arlaud G.J., Prohászka Z. (2007). Studies on the interactions between C-reactive protein and complement proteins. Immunology.

[18] Osmand A.P., Gerwurz H., Friedenson B. (1977). Partial amino-acid sequences of human and rabbit C-reactive proteins: Homology with immunoglobulins and histocompatibility antigens. Proc. Natl. Acad. Sci. USA.

[19] Pepys M.B. (2018). The pentraxins 1975–2018: Serendipity, diagnostics and drugs. Front Immunol.

[20] Gotschlich E.C., Edelman G.M. (1965). C-reactive protein: A molecule composed of subunits. Proc. Natl. Acad. Sci. USA.

[21] Szalai A.J., Agrawal A., Greenhough T.J., Volanakis J.E. (1999). C-reactive protein: Structural biology and host defense function. Clin. Chem. Lab. Med..

[22] Bang R., Marnell L., Mold C., Stein M.P., Clos K.T., Chivington-Buck C., Clos T.W. (2005). Analysis of binding sites in human C-reactive protein for FcγRI, FcγRIIA, and C1q by site-directed mutagenesis. J. Biol. Chem..

[23] Bally I., Ancelet S., Moriscot C., Gonnet F., Mantovani A., Daniel R., Schoehn G., Arlaud G.J., Thielens N.M. (2013). Expression of recombinant human complement C1q allows identification of the C1r/C1s-binding sites. Proc. Natl. Acad. Sci. USA.

[24] McCarthy M.I. (1947). The occurrence during acute infections of a protein not normally present in the blood. J. Exp. Med..

[25] DeLucas L.J., Greenhough T.J., Rule S.A., Myles D.A., Babu Y.S., Volanakis J.E., Bugg C.E. (1987). Preliminary X-ray study of crystals of human C-reactive protein. J. Mol. Biol..

[26] Ramadan M.A., Shrive A.K., Holden D., Myles D.A., Volanakis J.E., DeLucas L.J., Greenhough T.J. (2002). The three-dimensional structure of calcium-depleted human C-reactive protein from perfectly twinned crystals. Acta Crystallogr. D Biol. Crystallogr..

[27] Potempa L.A., Maldonado B.A., Laurent P., Zemel E.S., Gewurz H. (1983). Antigenic, electrophoretic and binding alterations of human C-reactive protein modified selectively in the absence of calcium. Mol. Immunol..

[28] Wang M.Y., Ji S.R., Bai C.J., El Kebir D., Li H.Y., Shi J.M., Zhu W., Costantino S., Zhou H.H., Potempa L.A. (2011). A redox switch in C-reactive protein modulates activation of endothelial cells. FASEB J..

[29] Du Clos T.W. (2013). Pentraxins: Structure, function, and role in inflammation. ISRN Inflamm.

[30] Black S., Kushner I., Samols D. (2004). C-reactive Protein. J. Biol. Chem..

[31] Agassandian M., Shurin G.V., Ma Y., Shurin M.R. (2014). C-reactive protein and lung diseases. Int. J. Biochem. Cell Biol..

[32] Pepys M.B., Hirschfield G.M. (2003). C-reactive protein: A critical update. J. Clin. Invest..

[33] Karasahin O., Tasar P.T., Timur O., Yildirim F., Binici D.N., Sahin S. (2018). The value of C-reactive protein in infection diagnosis and prognosis in elderly patients. Aging Clin. Exp. Res..

[34] Ryu J.A., Yang J.H., Lee D., Park C.M., Suh G.Y., Jeon K., Cho J., Baek S.Y., Carriere K.C., Chung C.R. (2015). Clinical Usefulness of Procalcitonin and C-Reactive Protein as Outcome Predictors in Critically Ill Patients with Severe Sepsis and Septic Shock. PLoS ONE.

[35] Bray C., Bell L.N., Liang H., Haykal R., Kaiksow F., Mazza J.J., Yale S.H. (2016). Erythrocyte Sedimentation Rate and C-reactive Protein Measurements and Their Relevance in Clinical Medicine. Wis. Med. J..

[36] Ticinesi A., Lauretani F., Nouvenne A., Porro E., Fanelli G., Maggio M., Meschi T. (2017). C-reactive protein (CRP) measurement in geriatric patients hospitalized for acute infection. Eur. J. Intern. Med..

[37] Vermeire S., Van Assche G., Rutgeerts P. (2005). The role of C-reactive protein as an inflammatory marker in gastrointestinal diseases. Nat. Clin. Pract. Gastr..

[38] Sproston N.R., Ashworth J.J. (2018). Role of C-Reactive Protein at Sites of Inflammation and Infection. Front. Immunol..

[39] Chandrashekara S. (2014). C-reactive protein: An inflammatory marker with specific role in physiology, pathology, and diagnosis. J. Rheumatol. Clin. Immunol..

[40] Simental-Mendia L.E., Sahebkar A., Rodriguez-Moran M., Zambrano-Galvan G., Guerrero-Romero F. (2017). Effect of Magnesium Supplementation on Plasma C-reactive Protein Concentrations: A Systematic Review and Meta-Analysis of Randomized Controlled Trials. Curr. Pharm. Des..

[41] Tang Y., Fung E., Xu A., Lan H.Y. (2017). C-reactive protein and ageing. Clin. Exp. Pharmacol. Physiol..

[42] Lu J., Mold C., Du Clos T.W., Sun P.D. (2018). Pentraxins and Fc Receptor-Mediated Immune Responses. Front. Immunol..

[43] Pepys M.B. (2005). CRP or not CRP? That Is the Question. Arterioscler Thromb. Vasc. Biol..

[44] Agrawal A., Gang T.B., Rusinol A.E. (2014). Recognition functions of pentameric C-reactive protein in cardiovascular disease. Mediators Inflamm..

[45] Ji S.R., Wu Y., Potempa L.A., Qiu Q., Zhao J. (2006). Interactions of C-reactive protein with low-density lipoproteins: Implications for an active role of modified C-reactive protein in atherosclerosis. Int. J. Biochem. Cell Biol..

[46] Khreiss T., Jozsef L., Potempa L.A., Filep J.G. (2004). Conformational rearrangement in C-reactive protein is required for proinflammatory actions on human endothelial cells. Circulation.

[47] Pepys M.B., Hawkins P.N., Kahan M.C., Tennent G.A., Gallimore J.R., Graham D., Sabin C.A., Zychlinsky A., De Diego J. (2005). Pro-inflammatory Effects of Bacterial Recombinant Human C-Reactive Protein are Caused by Contamination with Bacterial Products not by C-Reactive Protein Itself. Circ. Res..

[48] Molins B., Pena E., de la Torre R., Badimon L. (2011). Monomeric C-reactive protein is prothrombotic and dissociates from circulating pentameric C-reactive protein on adhered activated platelets under flow. Cardiovasc. Res..

[49] Heuertz R.M., Schneider G.P., Potempa L.A., Webster R.O. (2005). Native and modified C-reactive protein bind different receptors on human neutrophils. Int. J. Biochem. Cell Biol..

[50] Crossman D.C., Morton A.C., Gunn J.P., Greenwood J.P., Hall A.S., Fox K.A., Lucking A.J., Flather M.D., Lees B., Foley C.E. (2008). Investigation of the effect of Interleukin-1 receptor antagonist (IL-1ra) on markers of inflammation in non-ST elevation acute coronary syndromes (The MRC-ILA-HEART Study). Trials.

[51] Galea J., Ogungbenro K., Hulme S., Patel H., Scarth S., Hoadley M., Illingworth K., McMahon C.J., Tzerakis N., King A.T. (2018). Reduction of inflammation after administration of interleukin-1 receptor antagonist following aneurysmal subarachnoid hemorrhage: Results of the Subcutaneous Interleukin-1Ra in SAH (SCIL-SAH) study. J. Neurosurg..

[52] Tilg H., Vannier E., Vachino G., Dinarello C.A., Mier J.W. (1993). Antiinflammatory Properties of Hepatic Acute Phase Proteins: Preferential Induction of Interleukin 1 (IL-1) Receptor Antagonist over ID113 Synthesis by Human Peripheral Blood Mononuclear Cells. J. Exp. Med..

[53] Mold C., Rodriguez W., Rodic-Polic B., Du Clos T.W. (2002). C-Reactive Protein Mediates Protection from Lipopolysaccharide Through Interactions With FcγR. J. Immunol..

[54] Filep J., Foldes-Filep E. (1989). Effects of C-reactive protein on human neutrophil granulocytes challenged with N-formyl-methionyl-leucyl-phenylalanine and platelet-activating factor. Life Sci..

[55] Heuertz R.M., Ahmed N., Webster R.O. (1996). Peptides derived from C-reactive protein inhibit neutrophil alveolitis. J. Immunol..

[56] Heuertz R.M., Tricomi S.M., Ezekiel U.R., Webster R.O. (1999). C-reactive protein inhibits chemotactic peptide-induced p38 mitogen-activated protein kinase activity and human neutrophil movement. J. Biol. Chem..

[57] Kew R.R., Hyers T.M., Webster R.O. (1990). Human C-reactive protein inhibits neutrophil chemotaxis in vitro: Possible implications for the adult respiratory distress syndrome. J. Lab. Clin. Med..

[58] Ling M.R., Chapple I.L., Creese A.J., Matthews J.B. (2014). Effects of C-reactive protein on the neutrophil respiratory burst in vitro. Innate Immun..

[59] Devaraj S., Dasu M.R., Singh U., Rao L.V., Jialal I. (2009). C-reactive protein stimulates superoxide anion release and tissue factor activity in vivo. Atherosclerosis.

[60] Noren Hooten N., Ejiogu N., Zonderman A.B., Evans M.K. (2012). Association of oxidative DNA damage and C-reactive protein in women at risk for cardiovascular disease. Arterioscler Thromb. Vasc. Biol..

[61] Mortensen R.F., Zhong W. (2000). Regulation of phagocytic leukocyte activities by C-reactive protein. J. Leukoc. Biol..

[62] Cheryk L.A., Hayes M.A., Gentry P.A. (1996). Modulation of bovine platelet function by C-reactive protein. Vet. Immunol. Immunopathol..

[63] Fiedel B.A., Gewurz H. (1976). Effects of C-reactive protein on platelet function. I. Inhibition of platelet aggregation and release reactions. J. Immunol..

[64] Fiedel B.A., Gewurz H. (1976). Effects of C-reactive protein on platelet function. II. Inhibition by CRP of platelet reactivities stimulated by poly-L-lysine, ADP, epinephrine, and collagen. J. Immunol..

[65] Filep J.G., Herman F., Kelemen E., Foldes-Filep E. (1991). C-reactive protein inhibits binding of platelet-activating factor to human platelets. Thromb. Res..

[66] Kilpatrick J.M., Virella G. (1985). Inhibition of platelet-activating factor by rabbit C-reactive protein. Clin. Immunol. Immunopathol..

[67] Sestito A., Sgueglia G.A., Spinelli A., Navarese E.P., Infusino F., Crea F., Lanza G.A. (2006). Increased platelet reactivity in unstable angina patients is not related to C-reactive protein levels. Platelets.

[68] Boncler M., Luzak B., Rozalski M., Golanski J., Rychlik B., Watala C. (2007). Acetylsalicylic acid is compounding to antiplatelet effect of C-reactive protein. Thromb. Res..

[69] Spieker L.E., Flammer A.J., Amacker N., Sudano I., Badimon J.J., Ruschitzka F., Luscher T.F., Noll G., Corti R. (2006). C-reactive protein influences shear stress-dependent platelet adhesion in patients with familiar hypercholesterolemia and coronary artery disease undergoing LDL apheresis. Thromb. Haemost..

[70] Molins B., Pena E., Vilahur G., Mendieta C., Slevin M., Badimon L. (2008). C-reactive protein isoforms differ in their effects on thrombus growth. Arterioscler. Thromb. Vasc. Biol..

[71] Grad E., Pachino R.M., Danenberg H.D. (2011). Endothelial C-reactive protein increases platelet adhesion under flow conditions. Am. J. Physiol. Heart Circ. Physiol..

[72] Yaron G., Brill A., Dashevsky O., Yosef-Levi I.M., Grad E., Danenberg H.D., Varon D. (2006). C-reactive protein promotes platelet adhesion to endothelial cells: A potential pathway in atherothrombosis. Br. J. Haematol..

[73] Boncler M., Rywaniak J., Sicinska P., Watala C. (2011). Effectiveness of modified C-reactive protein in the modulation of platelet function under different experimental conditions. Blood Coagul Fibrinolysis.

[74] Boncler M., Rywaniak J., Szymanski J., Potempa L.A., Rychlik B., Watala C. (2011). Modified C-reactive protein interacts with platelet glycoprotein Ib alpha. Pharmacol. Rep..

[75] Skoglund C., Wettero J., Skogh T., Sjowall C., Tengvall P., Bengtsson T. (2008). C-reactive protein and C1q regulate platelet adhesion and activation on adsorbed immunoglobulin G and albumin. Immunol. Cell Biol..

[76] Pasceri V., Willerson J.T., Yeh E.T. (2000). Direct proinflammatory effect of C-reactive protein on human endothelial cells. Circulation.

[77] Pasceri V., Cheng J.S., Willerson J.T., Yeh E.T. (2001). Modulation of C-reactive protein-mediated monocyte chemoattractant protein-1 induction in human endothelial cells by anti-atherosclerosis drugs. Circulation.

[78] Venugopal S.K., Devaraj S., Yuhanna I., Shaul P., Jialal I. (2002). Demonstration that C-reactive protein decreases eNOS expression and bioactivity in human aortic endothelial cells. Circulation.

[79] Verma S., Wang C.H., Li S.H., Dumont A.S., Fedak P.W., Badiwala M.V., Dhillon B., Weisel R.D., Li R.K., Mickle D.A. (2002). A self-fulfilling prophecy: C-reactive protein attenuates nitric oxide production and inhibits angiogenesis. Circulation.

[80] Grad E., Danenberg H.D. (2013). C-reactive protein and atherothrombosis: Cause or effect?. Blood Rev..

[81] Tvarijonaviciute A., Aznar-Cayuela C., Rubio C.P., Ceron J.J., López-Jornet P. (2016). Evaluation of salivary oxidate stress biomarkers, nitric oxide and C-reactive protein in patients with oral lichen planus and burning mouth syndrome. J. Oral. Pathol. Med..

[82] Fichtlscherer S., Rosenberger G., Walter D.H., Breuer S., Dimmeler S., Zeiher A.M. (2000). Elevated C-reactive protein levels and impaired endothelial vasoreactivity in patients with coronary artery disease. Circulation.

[83] Nabata A., Kuroki M., Ueba H., Hashimoto S., Umemoto T., Wada H., Yasu T., Saito M., Momomura S., Kawakami M. (2008). C-reactive protein induces endothelial cell apoptosis and matrix metalloproteinase-9 production in human mononuclear cells: Implications for the destabilization of atherosclerotic plaque. Atherosclerosis.

[84] Venugopal S.K., Devaraj S., Jialal I. (2003). C-reactive protein decreases prostacyclin release from human aortic endothelial cells. Circulation.

[85] Hein T.W., Qamirani E., Ren Y., Kuo L. (2009). C-reactive protein impairs coronary arteriolar dilation to prostacyclin synthase activation: Role of peroxynitrite. J. Mol. Cell Cardiol..

[86] Devaraj S., Xu D.Y., Jialal I. (2003). C-reactive protein increases plasminogen activator inhibitor-1 expression and activity in human aortic endothelial cells: Implications for the metabolic syndrome and atherothrombosis. Circulation.

[87] Ji Y., Fish P.M., Strawn T.L., Lohman A.W., Wu J., Szalai A.J., Fay W.P. (2014). C-reactive protein induces expression of tissue factor and plasminogen activator inhibitor-1 and promotes fibrin accumulation in vein grafts. J. Thromb. Haemost..

[88] Singh U., Devaraj S., Jialal I. (2005). C-Reactive Protein Decreases Tissue Plasminogen Activator Activity in Human Aortic Endothelial Cells Evidence that C-Reactive Protein Is a Procoagulant. Arterioscler. Thromb. Vasc. Biol..

[89] Cermak J., Key N.S., Bach R.R., Balla J., Jacob H.S., Vercellotti G.M. (1993). C-Reactive Protein Induces Human Peripheral Blood Monocytes to Synthesize Tissue Factor. Blood.

[90] Cirillo P., Golino P., Calabro P., Calì G., Ragni M., De Rosa S., Cimmino G., Pacileo M., De Palma R., Forte L. (2005). C-reactive protein induces tissue factor expression and promotes smooth muscle and endothelial cell proliferation. Cardiovasc. Res..

[91] Paffen E., Vos H.L., Bertina R.M. (2004). C-Reactive Protein Does Not Directly Induce Tissue Factor in Human Monocytes. Arterioscler. Thromb. Vasc. Biol..

[92] Mousa S.A. (2006). Inhibitory effect of C-reactive protein on the release of tissue factor pathway inhibitor from human endothelial cells: Reversal by low molecular weight heparin. Int. Angiol..

[93] Li L., Roumeliotis N., Sawamura T., Renier G. (2004). C-reactive protein enhances LOX-1 expression in human aortic endothelial cells: Relevance of LOX-1 to C-reactive protein-induced endothelial dysfunction. Circ. Res..

[94] Wang C.H., Li S.H., Weisel R.D., Fedak P.W., Dumont A.S., Szmitko P., Li R.K., Mickle D.A., Verma S. (2003). C-reactive protein upregulates angiotensin type 1 receptors in vascular smooth muscle. Circulation.

[95] Devaraj S., Yun J.M., Adamson G., Galvez J., Jialal I. (2009). C-reactive protein impairs the endothelial glycocalyx resulting in endothelial dysfunction. Cardiovasc. Res..

[96] Bisoendial R.J., Kastelein J.J., Levels J.H., Zwaginga J.J., van den Bogaard B., Reitsma P.H., Meijers J.C., Hartman D., Levi M., Stroes E.S. (2005). Activation of Inflammation and Coagulation After Infusion of C-Reactive Protein in Humans. Circ. Res..

[97] Bisoendial R.J., Kastelein J.J., Peters S.L., Levels J.H., Birjmohun R., Rotmans J.I., Hartman D., Meijers J.C., Levi M., Stroes E.S. (2007). Effects of CRP infusion on endothelial function and coagulation in normocholesterolemic and hypercholesterolemic subjects. J. Lipid Res..

[98] Xu P.C., Lin S., Yang X.W., Gu D.M., Yan T.K., Wei L., Wang B.L. (2015). C-reactive protein enhances activation of coagulation system and inflammatory response through dissociating into monomeric form in antineutrophil cytoplasmic antibody-associated vasculitis. BMC Immunol..

[99] Biggs T.C., Bird J.H., Frampton S.J., Harries P.G., Salib R.J. (2014). C-reactive protein and coagulation studies in secondary post-tonsillectomy haemorrhage—Need for routine testing? Our experience in ninety-three patients. Clin. Otolaryngol..

[100] Pepys M.B., Hirschfield G.M., Tennent G.A., Gallimore J.R., Kahan M.C., Bellotti V., Hawkins P.N., Myers R.M., Smith M.D., Polara A. (2006). Targeting C-reactive protein for the treatment of cardiovascular disease. Nature.

[101] Thiele J.R., Habersberger J., Braig D., Schmidt Y., Goerendt K., Maurer V., Bannasch H., Scheichl A., Woollard K.J., von Dobschutz E. (2014). Dissociation of pentameric to monomeric C-reactive protein localizes and aggravates inflammation: In vivo proof of a powerful proinflammatory mechanism and a new anti-inflammatory strategy. Circulation.

[102] Slevin M., Iemma R.S., Zeinolabediny Y., Liu D., Ferris G.R., Caprio V., Phillips N., Di Napoli M., Guo B., Zeng X. (2018). Acetylcholine Inhibits Monomeric C-Reactive Protein Induced Inflammation, Endothelial Cell Adhesion, and Platelet Aggregation; A Potential Therapeutic?. Front. Immunol..

[103] Hounsell E.F., Davies M.J., Renouf D.V. (1996). O-linked protein glycosylation structure and function. Glycoconj. J..

[104] Spiro R.G. (2002). Protein glycosylation: Nature, distribution, enzymatic formation, and disease implications of glycopeptide bonds. Glycobiology.

[105] Sola R.J., Griebenow K. (2010). Glycosylation of therapeutic proteins: An effective strategy to optimize efficacy. BioDrugs.

[106] Goettig P. (2016). Effects of Glycosylation on the Enzymatic Activity and Mechanisms of Proteases. Int. J. Mol. Sci..

[107] Ohtsubo K., Marth J.D. (2006). Glycosylation in cellular mechanisms of health and disease. Cell.

[108] Gornik O., Lauc G. (2008). Glycosylation of serum proteins in inflammatory diseases. Dis. Markers.

[109] Das T., Sen A.K., Kempf T., Pramanik S.R., Mandal C., Mandal C. (2003). Induction of glycosylation in human C-reactive protein under different pathological conditions. Biochem. J..

[110] Vigushin D.M., Pepys M.B., Hawkins P.N. (1993). Metabolic and scintigraphic studies of radioiodinated human C-reactive protein in health and disease. J. Clin. Invest..

[111] Das T., Mandal C., Mandal C. (2004). Variations in binding characteristics of glycosylated human C-reactive proteins in different pathological conditions. Glycoconj. J..

[112] Ansar W., Mukhopadhyay S., Habib S.K., Basu S., Saha B., Sen A.K., Mandal C.N., Mandal C. (2009). Disease-associated glycosylated molecular variants of human C-reactive protein activate complement-mediated hemolysis of erythrocytes in tuberculosis and Indian visceral leishmaniasis. Glycoconj. J..

[113] Wang H.W., Wu Y., Chen Y., Sui S.F. (2002). Polymorphism of structural forms of C-reactive protein. Int. J. Mol. Med..

[114] Blizniukov O.P., Kozmin L.D., Falikova V.V., Martynov A.I., Tischenko V.M. (2003). Effect of Calcium Ions on Hydrodynamic Properties of Pentameric and Decameric C-Reactive Protein in Solution. Mol. Biol. (Mosk).

[115] Okemefuna A.I., Stach L., Rana S., Buetas A.J., Gor J., Perkins S.J. (2010). C-reactive protein exists in an NaCl concentration-dependent pentamer-decamer equilibrium in physiological buffer. J. Biol. Chem..

[116] Li Q., Xu W., Xue X., Wang Q., Han L., Li W., Lv S., Liu D., Richards J., Shen Z. (2016). Presence of multimeric isoforms of human C-reactive protein in tissues and blood. Mol. Med. Rep..

[117] Wu Y., Ji S.R., Wang H.W., Sui S.F. (2002). Study of the spontaneous dissociation of rabbit C-reactive protein. Biochemistry (Mosc).

[118] Potempa L.A., Siegel J.N., Fiedel B.A., Potempa R.T., Gewurz H. (1987). Expression, detection and assay of a neoantigen (Neo-CRP) associated with a free, human C-reactive protein subunit. Mol. Immunol..

[119] Sui S.F., Liu Z., Li W., Xiao C., Wang S., Gao Q., Zhou Q. (1996). Two-dimensional crystallization of rabbit C-reactive protein on lipid monolayers. FEBS Lett..

[120] Wang H.W., Sui S.F. (2001). Dissociation and subunit rearrangement of membrane-bound human C-reactive proteins. Biochem. Biophys. Res. Commun..

[121] Wu Y., Wang H.W., Ji S.R., Sui S.F. (2003). Two-dimensional crystallization of rabbit C-­reactive protein monomeric subunits. Acta Crystallogr. D Biol. Crystallogr..

[122] Ji S.R., Wu Y., Zhu L., Potempa L.A., Sheng F.L., Lu W., Zhao J. (2007). Cell membranes and liposomes dissociate C-reactive protein (CRP) to form a new, biologically active structural intermediate: mCRP(m). FASEB J..

[123] de la Torre R., Pena E., Vilahur G., Slevin M., Badimon L. (2013). Monomerization of C-reactive protein requires glycoprotein IIb-IIIa activation: Pentraxins and platelet deposition. J. Thromb. Haemost..

[124] Eisenhardt S.U., Habersberger J., Murphy A., Chen Y.C., Woollard K.J., Bassler N., Qian H., von Zur Muhlen C., Hagemeyer C.E., Ahrens I. (2009). Dissociation of pentameric to monomeric C-reactive protein on activated platelets localizes inflammation to atherosclerotic plaques. Circ. Res..

[125] Braig D., Kaiser B., Thiele J.R., Bannasch H., Peter K., Stark G.B., Koch H.G., Eisenhardt S.U. (2014). A conformational change of C-reactive protein in burn wounds unmasks its proinflammatory properties. Int. Immunol..

[126] Lauer N., Mihlan M., Hartmann A., Schlotzer-Schrehardt U., Keilhauer C., Scholl H.P., Charbel Issa P., Holz F., Weber B.H., Skerka C. (2011). Complement regulation at necrotic cell lesions is impaired by the age-related macular degeneration-associated factor-H His402 risk variant. J. Immunol..

[127] Mihlan M., Blom A.M., Kupreishvili K., Lauer N., Stelzner K., Bergstrom F., Niessen H.W., Zipfel P.F. (2011). Monomeric C-reactive protein modulates classic complement activation on necrotic cells. FASEB J..

[128] Hammond D.J. Jr., Singh S.K., Thompson J.A., Beeler B.W., Rusinol A.E., Pangburn M.K., Potempa L.A., Agrawal A. (2010). Identification of acidic pH-dependent ligands of pentameric C-reactive protein. J. Biol. Chem..

[129] Boncler M., Kehrel B., Szewczyk R., Stec-Martyna E., Bednarek R., Brodde M., Watala C. (2018). Oxidation of C-reactive protein by hypochlorous acid leads to the formation of potent platelet activator. Int. J. Biol. Macromol..

[130] Singh S.K., Thirumalai A., Pathak A., Ngwa D.N., Agrawal A. (2017). Functional Transformation of C-reactive Protein by Hydrogen Peroxide. J. Biol. Chem..

[131] Habersberger J., Strang F., Scheichl A., Htun N., Bassler N., Merivirta R.M., Diehl P., Krippner G., Meikle P., Eisenhardt S.U. (2012). Circulating microparticles generate and transport monomeric C-reactive protein in patients with myocardial infarction. Cardiovasc. Res..

[132] Strang F., Scheichl A., Chen Y.C., Wang X., Htun N.M., Bassler N., Eisenhardt S.U., Habersberger J., Peter K. (2012). Amyloid plaques dissociate pentameric to monomeric C-reactive protein: A novel pathomechanism driving cortical inflammation in Alzheimer’s disease?. Brain Pathol..

[133] Ji S.R., Wu Y., Potempa L.A., Liang Y.H., Zhao J. (2006). Effect of modified C-reactive protein on complement activation: A possible complement regulatory role of modified or monomeric C-reactive protein in atherosclerotic lesions. Arterioscler. Thromb. Vasc. Biol..

[134] Mihlan M., Stippa S., Jozsi M., Zipfel P.F. (2009). Monomeric CRP contributes to complement control in fluid phase and on cellular surfaces and increases phagocytosis by recruiting factor H. Cell Death Differ..

[135] Ji S.R., Ma L., Bai C.J., Shi J.M., Li H.Y., Potempa L.A., Filep J.G., Zhao J., Wu Y. (2009). Monomeric C-reactive protein activates endothelial cells via interaction with lipid raft microdomains. FASEB J..

[136] Khreiss T., Jozsef L., Hossain S., Chan J.S., Potempa L.A., Filep J.G. (2002). Loss of pentameric symmetry of C-reactive protein is associated with delayed apoptosis of human neutrophils. J. Biol. Chem..

[137] Khreiss T., Jozsef L., Potempa L.A., Filep J.G. (2004). Opposing effects of C-reactive protein isoforms on shear-induced neutrophil-platelet adhesion and neutrophil aggregation in whole blood. Circulation.

[138] Khreiss T., Jozsef L., Potempa L.A., Filep J.G. (2005). Loss of pentameric symmetry in C-reactive protein induces interleukin-8 secretion through peroxynitrite signaling in human neutrophils. Circ. Res..

[139] Zouki C., Haas B., Chan J.S., Potempa L.A., Filep J.G. (2001). Loss of pentameric symmetry of C-reactive protein is associated with promotion of neutrophil-endothelial cell adhesion. J. Immunol..

[140] Li H.Y., Wang J., Meng F., Jia Z.K., Su Y., Bai Q.F., Lv L.L., Ma F.R., Potempa L.A., Yan Y.B. (2016). An Intrinsically Disordered Motif Mediates Diverse Actions of Monomeric C-reactive Protein. J. Biol. Chem..

[141] Schwedler S.B., Guderian F., Dammrich J., Potempa L.A., Wanner C. (2003). Tubular staining of modified C-reactive protein in diabetic chronic kidney disease. Nephrol. Dial. Transplant..

[142] Crawford J.R., Trial J., Nambi V., Hoogeveen R.C., Taffet G.E., Entman M.L. (2016). Plasma Levels of Endothelial Microparticles Bearing Monomeric C-reactive Protein are Increased in Peripheral Artery Disease. J. Cardiovasc. Transl. Res..

[143] Hu W.P., Hsu H.Y., Chiou A., Tseng K.Y., Lin H.Y., Chang G.L., Chen S.J. (2006). Immunodetection of pentamer and modified C-reactive protein using surface plasmon resonance biosensing. Biosens. Bioelectron..

[144] Ying S.C., Gewurz H., Kinoshita C.M., Potempa L.A., Siegel J.N. (1989). Identification and partial characterization of multiple native and neoantigenic epitopes of human C-reactive protein by using monoclonal antibodies. J. Immunol..

[145] Ying S.C., Shephard E., de Beer F.C., Siegel J.N., Harris D., Gewurz B.E., Fridkin M., Gewurz H. (1992). Localization of sequence-determined neoepitopes and neutrophil digestion fragments of C-reactive protein utilizing monoclonal antibodies and synthetic peptides. Mol. Immunol..

[146] Bell S.A., Faust H., Schmid A., Meurer M. (1998). Autoantibodies to C-reactive protein (CRP) and other acute-phase proteins in systemic autoimmune diseases. Clin. Exp. Immunol..

[147] Sjowall C., Eriksson P., Almer S., Skogh T. (2002). Autoantibodies to C-reactive protein is a common finding in SLE, but not in primary Sjögren’s syndrome, rheumatoid arthritis or inflammatory bowel disease. J. Autoimmun..

[148] Tan Y., Yu F., Yang H., Chen M., Fang Q., Zhao M.H. (2008). Autoantibodies against monomeric C-reactive protein in sera from patients with lupus nephritis are associated with disease activity and renal tubulointerstitial lesions. Hum. Immunol..

[149] Li Q.Y., Li H.Y., Fu G., Yu F., Wu Y., Zhao M.H. (2017). Autoantibodies against C-Reactive Protein Influence Complement Activation and Clinical Course in Lupus Nephritis. J. Am. Soc. Nephrol..

[150] Hirschfield G.M., Gallimore J.R., Kahan M.C., Hutchinson W.L., Sabin C.A., Benson G.M., Dhillon A.P., Tennent G.A., Pepys M.B. (2005). Transgenic human C-reactive protein is not proatherogenic in apolipoprotein E-deficient mice. Proc. Natl. Acad. Sci. USA.

[151] Koike T., Kitajima S., Yu Y., Nishijima K., Zhang J., Ozaki Y., Morimoto M., Watanabe T., Bhakdi S., Asada Y. (2009). Human C-reactive protein does not promote atherosclerosis in transgenic rabbits. Circulation.

[152] Kovacs A., Tornvall P., Nilsson R., Tegner J., Hamsten A., Bjorkegren J. (2007). Human C-reactive protein slows atherosclerosis development in a mouse model with human-like hypercholesterolemia. Proc. Natl. Acad. Sci. USA.

[153] Paul A., Ko K.W., Li L., Yechoor V., McCrory M.A., Szalai A.J., Chan L. (2004). C-reactive protein accelerates the progression of atherosclerosis in apolipoprotein E-deficient mice. Circulation.

[154] Reifenberg K., Lehr H.A., Baskal D., Wiese E., Schaefer S.C., Black S., Samols D., Torzewski M., Lackner K.J., Husmann M. (2005). Role of C-reactive protein in atherogenesis: Can the apolipoprotein E knockout mouse provide the answer?. Arterioscler. Thromb. Vasc. Biol..

[155] Teupser D., Weber O., Rao T.N., Sass K., Thiery J., Fehling H.J. (2011). No reduction of atherosclerosis in C-reactive protein (CRP)-deficient mice. J. Biol. Chem..

[156] Trion A., de Maat M.P., Jukema J.W., van der Laarse A., Maas M.C., Offerman E.H., Havekes L.M., Szalai A.J., Princen H.M., Emeis J.J. (2005). No effect of C-reactive protein on early atherosclerosis development in apolipoprotein E*3-leiden/human C-reactive protein transgenic mice. Arterioscler. Thromb. Vasc. Biol..

[157] Elliott P., Chambers J.C., Zhang W., Clarke R., Hopewell J.C., Peden J.F., Erdmann J., Braund P., Engert J.C., Bennett D. (2009). Genetic Loci associated with C-reactive protein levels and risk of coronary heart disease. JAMA.

[158] Ridker P.M. (2016). From C-Reactive Protein to Interleukin-6 to Interleukin-1: Moving Upstream To Identify Novel Targets for Atheroprotection. Circ. Res..

[159] Zacho J., Tybjaerg-Hansen A., Jensen J.S., Grande P., Sillesen H., Nordestgaard B.G. (2008). Genetically elevated C-reactive protein and ischemic vascular disease. N Engl. J. Med..

[160] Jia Z.K., Li H.Y., Liang Y.L., Potempa L.A., Ji S.R., Wu Y. (2018). Monomeric C-Reactive Protein Binds and Neutralizes Receptor Activator of NF-κB Ligand-Induced Osteoclast Differentiation. Front. Immunol..

[161] Wang J., Tang B., Liu X., Wu X., Wang H., Xu D., Guo Y. (2015). Increased monomeric CRP levels in acute myocardial infarction: A possible new and specific biomarker for diagnosis and severity assessment of disease. Atherosclerosis.

[162] Zhang L., Li H.Y., Li W., Shen Z.Y., Wang Y.D., Ji S.R., Wu Y. (2018). An ELISA Assay for Quantifying Monomeric C-Reactive Protein in Plasma. Front. Immunol..

[163] Lee J.W. (2009). Method validation and application of protein biomarkers: Basic similarities and differences from biotherapeutics. Bioanalysis.

[164] Dasu M.R., Devaraj S., Du Clos T.W., Jialal I. (2007). The biological effects of CRP are not attributable to endotoxin contamination: Evidence from TLR4 knockdown human aortic endothelial cells. J. Lipid Res..

[165] Potempa L.A., Yao Z.Y., Ji S.R., Filep J.G., Wu Y. (2015). Solubilization and purification of recombinant modified C-reactive protein from inclusion bodies using reversible anhydride modification. Biophys. Rep..

[166] Taylor K.E., Giddings J.C., Van Den Berg C.W. (2005). C-Reactive Protein–Induced In Vitro Endothelial Cell Activation Is an Artefact Caused by Azide and Lipopolysaccharide. Arterioscler. Thromb. Vasc. Biol..

[167] Thiele J.R., Zeller J., Bannasch H., Stark G.B., Peter K., Eisenhardt S.U. (2015). Targeting C-Reactive Protein in Inflammatory Disease by Preventing Conformational Changes. Mediators Inflamm..

[168] Boguslawski G., McGlynn P.W., Potempa L.A., Filep J.G., Labarrere C.A. (2007). Conduct unbecoming: C-reactive protein interactions with a broad range of protein molecules. J. Heart Lung Transplant..

[169] Boncler M., Dudzinska D., Nowak J., Watala C. (2012). Modified C-reactive protein selectively binds to immunoglobulins. Scand. J. Immunol.

